# Supplementary material for: Sustenance Trial to Analyze the Effects of Black Soldier Fly Larvae Meal on the Reproductive Efficiency of Sows and the Hematological Properties of Suckling and Weaning Piglets
Source: Animals (Basel). 2023 Nov 3;13(21):3410. doi: 10.3390/ani13213410 (PMC10647472; doi:10.3390/ani13213410)
Supplement: Supplementary file 1 [file animals-13-03410-s001.zip › animals-2673398-supplementary.pdf]

Sustenance trial to analyze the effects of black soldier fly larvae meal on the reproductive efficiency of sows and the hematological properties of suckling and weaning piglets

Kiyonori Kawasaki [1,*], Junliang Zhao [1], Natsu Takao [1], Masaki Sato [1], Takuma Ban [1], Kaoru Tamamaki [1], Masanori Kagami [1], Kiminobu Yano [2]

[1] Department of Applied Biological Science, Faculty of Agriculture, Kagawa University, Ikenobe 2393, Miki-cho, Kita-gun, Kagawa 761-0795, Japan.

[2] University Farm, Kagawa University, Showa 300-2, Sanuki, Kagawa 769-2304, Japan.

* Correspondence: kawasaki.kiyonori@kagawa-u.ac.jp; +81-87-891-3061

Supplementary Data

Table S1. Parity number of the sows.

C		L		H	
Sow	Parity	Sow	Parity	Sow	Parity
C1	Gilt	L1	Gilt	H1	Gilt
C2	Gilt	L2	Gilt	H2	Gilt
C3	Gilt	L3	Gilt	H3	Gilt
C4	Gilt	L4	Gilt	H4	Gilt
C5	Gilt	L5	Gilt	H5	Gilt
C6	Gilt	L6	1	H6	1
C7	1	L7	1	H7	1
C8	1	L8	1	H8	1
C9	1	L9	1	H9	1
C10	2	L10	2	H10	1

Table S2. Body weight and sex of the weaned piglets in each pen.

C			L			H		
Pen	Sex	Body weight (kg)	Pen	Sex	Body weight (kg)	Pen	Sex	Body weight (kg)
C1	Barrow	10.46	L1	Barrow	9.70	H1	Barrow	8.69
	Barrow	9.79		Gilt	11.79		Barrow	10.16
	Barrow	8.42		Gilt	11.71		Barrow	10.54
	Gilt	10.01		Gilt	12.57		Barrow	9.57
	Gilt	8.26		Gilt	11.54		Barrow	10.09
	Gilt	8.93		Gilt	9.95		Gilt	8.34
C2	Barrow	9.24	L2	Barrow	9.63	H2	Barrow	10.21
	Barrow	8.28		Barrow	9.11		Barrow	9.50
	Barrow	8.09		Gilt	9.65		Barrow	8.51
	Gilt	11.67		Gilt	9.96		Gilt	6.31
	Gilt	12.92		Gilt	9.58		Gilt	8.56
	Gilt	9.25		Gilt	8.25		Gilt	9.58
C3	Barrow	8.29	L3	Barrow	13.59	H3	Barrow	8.53
	Barrow	7.68		Barrow	6.06		Barrow	8.37
	Barrow	6.94		Gilt	10.77		Barrow	6.98
	Gilt	9.79		Gilt	11.40		Gilt	8.77
	Gilt	7.29		Gilt	9.36		Gilt	6.52
	Gilt	7.61		Gilt	7.40		Gilt	7.16
C4	Barrow	7.00	L4	Barrow	7.14	H4	Barrow	7.29
	Barrow	10.29		Barrow	7.24		Barrow	8.57
	Barrow	8.71		Gilt	7.37		Barrow	9.01
	Barrow	8.41		Gilt	7.36		Barrow	8.94
	Barrow	8.30		Gilt	10.28		Barrow	8.08
	Gilt	7.61		Gilt	12.83		Barrow	7.63
C5	Barrow	7.86	L5	Barrow	12.28	H5	Barrow	6.75
	Barrow	7.94		Barrow	9.20		Barrow	7.42
	Gilt	8.73		Barrow	9.90		Barrow	8.47
	Gilt	7.60		Barrow	9.12		Barrow	8.63
	Gilt	8.83		Gilt	7.44		Barrow	8.90
	Gilt	7.71		Gilt	7.41		Barrow	8.94
